# Increased interferon alpha receptor 2 mRNA levels is associated with renal cell carcinoma metastasis

**DOI:** 10.1186/1471-2407-7-159

**Published:** 2007-08-15

**Authors:** Takao Kamai, Yoshiaki Yanai, Kyoko Arai, Hideyuki Abe, Tomonori Yamanishi, Masashi Kurimoto, Ken-Ichiro Yoshida

**Affiliations:** 1 Department of Urology, Dokkyo Medical University, Tochigi, Japan; 2 Institution of Fujisaki, Hayashibara Biochemical Lab., Inc, Okayama, Japan

## Abstract

**Background:**

Interferon-α (IFN-α) is one of the central agents in immunotherapy for renal cell carcinoma (RCC) and binds to the IFN-α receptor (IFNAR). We investigated the role of IFNAR in RCC.

**Methods:**

We quantified IFNAR mRNA expression in paired tumor and non-tumor samples from the surgical specimens of 103 consecutive patients with RCC using a real-time reverse transcription polymerase chain reaction (RT-PCR), and IFNAR2 protein using Western blotting.

**Results:**

The absolute level of IFNAR1 and IFNAR2 mRNAs in tumor and non-tumor tissues did not correlate with the malignant and metastatic profiles. The relative yields of the PCR product from the tumor tissue to that from the corresponding non-tumor tissue (T/N) for the expression of IFNAR mRNAs were calculated. While the T/N ratio of IFNAR1 did not correlate with any factor, a high T/N ratio of IFNAR2 correlated with poor differentiation (*P *< 0.05), local invasion (*P *< 0.001), and metastasis (*P *< 0.0001). By multivariate analysis, a high T/N ratio of IFNAR2 predicted a shortened overall survival in all cases (*P *< 0.05) and a shorter disease-free survival in those without metastasis (M0; 68 cases, *P *< 0.05). Impressively, patients with a poorer response to IFN-α treatment had a higher IFNAR2 T/N ratio than those who had a good response (P < 0.05). IFNAR2c protein expression was higher in the primary tumors in patients with metastases (M1; 35 cases) compared to those without ( P < 0.0001).

**Conclusion:**

IFNAR2 is associated with the progression of RCC.

## Background

It has been reported that the incidence of renal cell carcinoma (RCC) is increasing steadily [1]. Although surgical resection of the primary tumor remains the mainstay of therapy, RCC is characterized by a high frequency of metastases at diagnosis or relapse following nephrectomy. Patients with distant metastases have a poor prognosis, with a 5-year survival rate of less than 10% [1]. RCC is notoriously resistant to chemotherapy and radiotherapy [1-3]. Immunotherapy with interferon-α (IFN-α) achieves responses in 10% to 20% of patients with advanced RCC [1,4]. IFN-α binds to the cell surface IFN-α receptor (IFNAR), which is composed of two chains, a 110 KDa subunit (IFNAR1) and a 102 KDa subunit (IFNAR2) [5]. Additionally, three different forms of IFNAR2 have been reported: a 40 KDa soluble form designated IFNAR2a, a 55 KDa short form known as IFNAR2b, and IFNAR2c, a 102 KDa long form. These different forms are derived from alternative splicing of the same gene. The IFNAR2c mediates a biologic response when associated with IFNAR1 [5]. There have been reports regarding the relationship between IFNAR expression and human hepatocelluar carcinoma [6,7]; however, there have been no published reports on the role of IFNAR in human RCC. We compared IFNAR mRNA expression in RCC tissues with non-neoplastic portions of the same resected specimen using a real-time reverse transcription-polymerase chain reaction (RT-PCR), and IFNAR2 protein expression using Western blotting. The relationship between IFNAR expression and selected pathologic features of the tumors was examined. We also assessed whether IFNAR expression could predict the survival of the patients with these tumors, and compared its expression in patients treated with IFN-α who had a good or poor response to therapy. We postulated that this might predict recurrence.

## Methods

### Patients and tissue preparation

We studied 103 consecutive Japanese patients (71 men, 32 women), 33 to 81 years old (mean age 63.7 years), with newly diagnosed clear cell RCC, from 1998 to 2005. All patients routinely underwent imaging studies (CT and/or MRI) for preoperative staging prior to radical nephrectomy. The postoperative follow-up ranged from 2 to 75 months (median, 29 months). Patients underwent surgery before receiving any other therapy.

For every case, three different tumor sites and varying portions of the non-neoplastic kidney were resected for the study. The resected tissues were stored at -80°C as described previously [8-10]. Grading and staging were carried out according to the criteria of the TNM classification [11]. The study was conducted in accordance with the Helsinki Declaration. Institutional Review Board approval was obtained for this investigation. Each patient signed a consent form approved by the Committee on Human Rights in Research of our institution.

Systemic postoperative immunotherapy using IFN-α was usually administered for the patients with pT3/4 tumors, poor differentiation and/or other organ involvement. These patients received 3, 5, or 6 million units of natural human IFN-α intravenously or intramuscularly two or three times a week for 12 weeks to 6 months, or until tumor progression. The dosage of IFN-α was decreased for grade 3/4 toxicity.

### Real-time RT-PCR assay

Total RNA was purified from all 103 sets of tumor and non-tumor RCC tissue samples using the RNA preparation kit "High Pure RNA Kit" (Roche Diagnostic Ltd, Germany). Total RNA was used as a template for cDNA synthesis. A 100 μL reaction mixture containing 1 μg of random hexamers and 100 units of MMLV-reverse transcriptase was incubated at 25°C for 10 min, 42°C for 30 min, and then 99°C for 5 min. The expression profiles of the IFNAR1 and IFNAR2 genes were analyzed with an ABI PRISM 7700 Sequence Detection System (Applied Biosystems, Foster City, CA) using the SYBR Green method. The following specific primers were designed to amplify their respective genes in all of the primary carcinoma tissues after confirming their specificities; IFNAR1, sense; 5'-TGACCAGAAATGAACTGTGTCA-3', anti-sense; 5'-TTTAAATAGTTAAGAGCTTGCCCG-3'; IFNAR2, sense; 5'-GAAGGTGGTTAAGAACTGTGC-3', anti-sense; 5'-CCCGCTGAATCCTTCTAGGACGG-3'; β 2-microglobulin, sense; 5'-ACCCCCACTGAAAAAGATGA-3', anti-sense; 5'-ATCTTCAAACCTCCATGATG). A real-time RT-PCR assay was performed on a 25 μL reaction mixture containing 20 ng of sample cDNA, 100 nM sense primer, 100 nM anti-sense primer, and 12.5 μL of SYBR Green PCR Master Mix (Applied Biosystems). The PCR was carried out for 45 cycles of 95°C for 15 sec and 60°C for 1 min. To normalize the amplified products in each sample, we used β 2-microglobulin as a quantitative internal control [9,10]. A standard curve for each mRNA expression was generated using five-fold dilutions of a control RNA sample (25×, 5×, 1×, 0.2×, 0.04×). The mRNA expression levels of each of the 2 targeted genes were presented as a ratio to that of β 2-microglobulin, and the relative expression levels were calculated [9,10]. The mean values from the real-time RT-PCR data for the three samples of the resected tissues were used for the analysis according to a method described previously [9,10].

### Western blotting assay

Tissue samples were incubated in lysis buffer (50 mM Tris-HCl, 150 mM NaCl, 1% Nonidet P-40, 1 mg/mL aprotinin, and 100 mM Na_3_VO_4_) on ice for 30 min. The mixture was centrifuged for 30 min (15,000 rpm, 4°C) and the supernatant was collected for Western blotting analysis. Samples (50 μg/lane) were electrophoresed in a 10/20% polyacrylamide muliti-gel 20 (Daiichi Pure Chemicals Co., Ltd., Tokyo, Japan), and transferred to a polyvinylidine difluoride transfer membrane. Membranes were blocked in 20 mM Tris HCl (pH 7.5) containing 10%-Broke-Ace and then probed with anti-human IFNAR1 polyclonal antibody (ANOC 4867; a kind gift from Otsuka Pharmaceutical, Tokushima, Japan) or anti-human IFNAR2 polyclonal antibody (ANOC 10403; a kind gift from Otsuka Pharmaceutical, Tokushima, Japan) for 1 h at room temperature [12,13]. Next, the membranes were washed several times with 0.1% Triton TBS, and then incubated with anti-rabbit IgG-HRP antibody (1:1000 dilution; Sigma Chemical, St. Louis, MO) for 1 h. The reaction was visualized using the ECL system (Amersham Pharmacia Biotech UK Limited, Buckinghamshire, England). Hyperfilm ECL (Amersham Pharmacia Biotech) was exposed to the membrane, developed, and the intensities of the specific bands were calibrated by NIH image software. For the quantification of proteins, the relative amounts of IFNAR2 in the tumors were expressed as a ratio of the optical density of the bands from the tumor specimen to those from the corresponding normal tissue; the latter was set at 1.0 by densitometric analysis as described previously [8,14]. The mean values from three experiments were obtained for all the tumor and non-tumor tissues [8-10,14].

### Immunohistochemistry

Immunohistochemistry, using the same specific antibodies as employed for Western blotting [12,13], was performed in 8 tumors with distant metastases (M1) and 8 tumors without (M0) to support the data obtained by Western blotting as described previously [8].

#### Statistical analysis

The results of real-time RT-PCR and Western blotting were analyzed statistically using the Mann-Whitney *U *test for two groups as described previously [8,10], and the Kruskal-Wallis test for three groups. The expression levels of mRNAs for IFNAR, as well as tumor grade and stage, were assessed in terms of survival by the Cox proportional hazards model using univariate and multivariate analyses. The Kaplan-Meier method was used to estimate survival as a function of time, and survival differences were assessed by the log-rank test. *P *values less than 0.05 were considered significant. Data were analyzed using commercially available software.

## Results

### IFNAR mRNA and protein expression and pathologic characteristics

The clinicopathological data are summarized in Table 1. IFNAR1 and IFNAR2 mRNAs were detected in tumor and non-tumor kidney specimens. The absolute level of IFNAR mRNAs ranges wildly due to inter-individual variations. The absolute level of mRNA expression for IFNAR1 and IFNAR2 in the tumor and non-tumor tissues did not correlate with the malignant and the metastatic profiles of the RCC tumors. Since inter-individual variations in the expression of IFNAR mRNAs may be important, the relative yield of the PCR product from the tumor to that from the corresponding non-tumor tissue (T/N) for expression of IFNAR1 and IFNAR2 was calculated as previously [9,10].

**Table 1:** Clinicopathologic data of the patients

Grade	pT	Metastasis and Sarcomatoid component	IFNa treatment	Effect of treatment
		M0 (10 acinar)	IFNa (-) (10 pts)	NED : 10 pts
	pT1/2 (12 pts)			
Grade 1 (No. of 14 patients)		M1 (lung meta of 2 acinar)	IFNa (+) (2 pts)	CR/PR : 1 pt, PD : 1 pt
	pT3/4 (2 pts)	M0 (2 acinar)	IFNa (+) (2 pts)	NED : 2 pts
			IFNa (-) (35 pts)	NED : 32 pts, Meta : 3 pts
		M0 (39 acinar)		
	pT1/2 (45 pts)		IFNa (+) (4 pts)	NED : 3 pts, Meta : 1 pt
Grade 2 (No. of 57 patients)		M1 (lung meta of 6 acinar)	IFNa (+) (6 pts)	CR/PR : 2 pts, PD : 4 pts
			IFNa (-) (2 pts)	NED : 1 pt, Meta : 1 pt
		M0 (7 acinar)		
	pT3/4 (12 pts)		IFNa (+) (5 pts)	NED : 4 pts, Meta : 1 pt
		M1 (lung meta of 5 acinar)	IFNa (+) (5 pts)	CR/PR : 2 pts, PD : 3 pts
		M0 (5 acinar)	IFNa (+) (5 pts)	NED : 2 pts, Meta : 3 pts
	pT1/2 (15 pts)			
		M1 (lung meta of 5 acinar	IFNa (+) (5 pts)	CR/PR : 2 pts, PD : 3 pts
Grade 3 (No. of 32 patients)		other organs meta in addition to lung of 5 sarcomatoid)	other systemic therapies including IFNa	PD : 5 pts
		M0 (5 acinar)	IFNa (+) (5 pts)	NED : 3 pts, Meta : 2 pts
	pT3/4 (17 pts)			
		M1 (lung meta of 5 acinar	IFNa (+) (5 pts)	PD : 5 pts
		other organs meta in addition to lung of 7 sarcomatoid)	other systemic therapies including IFNa	PD : 7 pts

There was no relationship between the T/N ratio of IFNAR1 mRNA and grade (mean ± S.D., grade 1, 0.93 ± 0.35; grade 2, 1.06 ± 0.38; grade 3, 2.51 ± 1.72), stage (pT1-2, 0.63 ± 0.17; pT3-4, 2.79 ± 2.40), or presence of metastasis (M0, 1.38 ± 0.41; M1, 5.57 ± 3.86).

**Figure 1:**
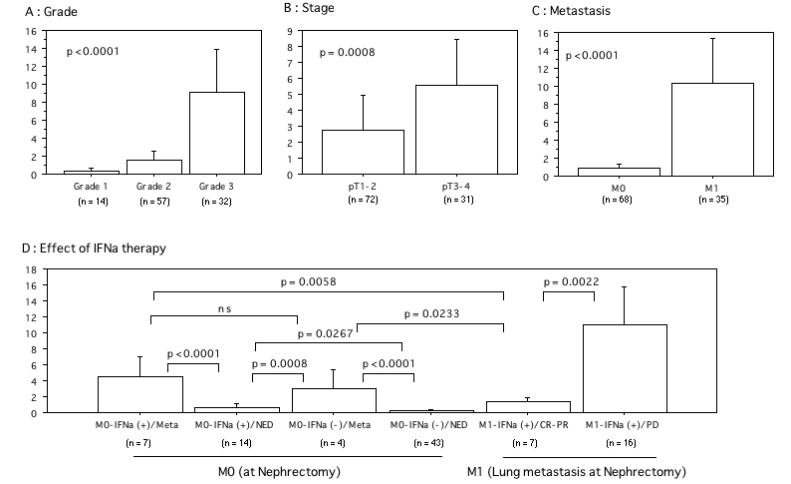
T (tumor)/N (non-tumor) values of IFNAR2 mRNA expression in kidney. A; In Grade 1 to 3 tumors. B; In pT1,2 and pT3,4 tumors. C; In metastasis (+) and (-). D; In effect of treatment of IFN-α (+) and watch-and-see (-). Meta; appearance of metastatic lesion after nephrectomy. NED; no evidence of disease. CR-PR; complete to partial response. PD; progression of disease. The data show the 95%

In contrast, a increased T/N ratio of IFNAR2 mRNA was associated with poorer differentiation: grade 1 tumors had a T/N = 0.32 ± 0.16; grade 2, 1.51 ± 0.54; and grade 3, 9.17 ± 2.31, *P *< 0.0001 (Figure 1A). The T/N ratio of IFNAR2 mRNA also correlated with stage: pT1-2 tumors had a T/N ratio = 2.75 ± 1.09, while the comparable value for pT3-4 tumors was 5.59 ± 1.41, *P *< 0.001 (Figure 1B). The T/N ratio of IFNAR2 was higher in M1 tumors compared to those in M0 tumors (10.36 ± 2.41 vs. 0.92 ± 0.23, *P *< 0.0001; Figure 1C).

The T/N ratio of IFNAR2 in tumors (that included a sarcomatoid component which confers a worse prognosis) was increased compared to the conventional acinar type (13.22 ± 4.89 vs. 2.19 ± 0.51, *P *< 0.0001). There was no statistical difference in the T/N ratio of IFNAR1 mRNA between sarcomatoid (4.35 ± 2.45) and conventional acinar type tumors (1.76 ± 0.78, P = 0.133), As shown in Table 1, 12 patients, including those with a sarcomatoid component (all patients with grade 3), presented with metastases at diagnosis.

**Figure 2:**
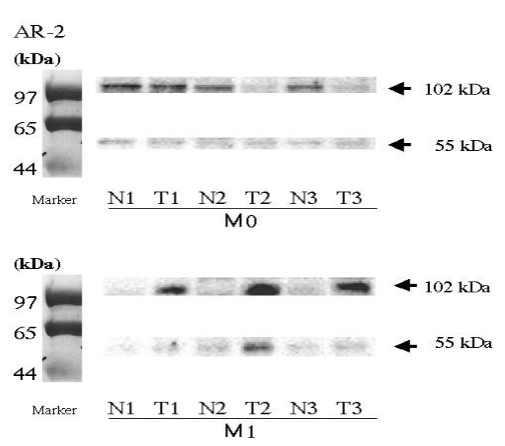
Western blotting analyses of IFNAR2 proteins in primary lesions with (M1) and without (M0) metastasis. IFNAR2b; 55KDa, IFNAR2c; 102 KDa. N; non-tumor, T; tumor. Each number corresponds to a case number.

For further characterization of the expression profiles of IFNAR2, we also analyzed the expression patterns of both the IFNAR2b (55 KDa) and IFNAR2c (102 KDa) proteins using Western blotting analysis (Figure 2). The results of these experiments revealed that the relative expression levels of IFNAR2b in tumors compared to the corresponding non-tumor sections, (set to 1.0), did not differ. There was no significant difference in the expression levels of IFNAR2c between tumor and non-tumor tissues in cases without metastasis (M0). Impressively, the IFNAR2c protein levels were significantly higher in the primary lesions with distant metastases (M1) (mean ± S.D., 11.29 ± 4.31, *P *< 0.0001).

**Figure 3:**
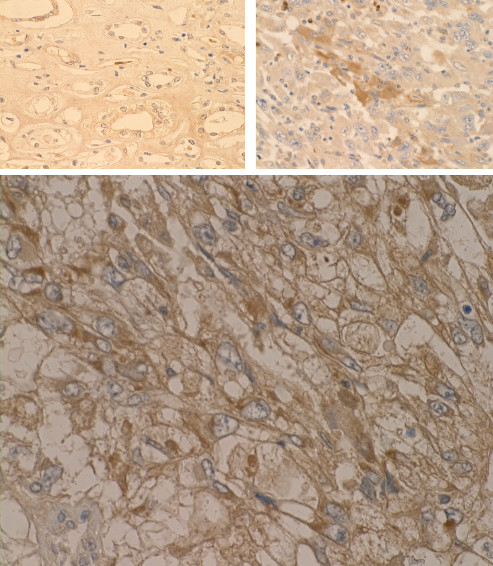
Immunohistochemistry for IFNAR2 proteins in M1 cases. Upper left shows negative staining in normal cells in M1 case (x50). Upper right shows strong brown staining in sarcomatoid carcinoma in M1 case (x50). Lower panel shows moderate brown staining in clear cell carcinoma with conventional acinar type in M1 case (x200).

While IFNAR2 proteins were moderately to intensely expressed in the cytoplasm of cancer cells in the M1 cases, these proteins were very weakly expressed by immunohistochemistry in normal cells in M1 cases (Figure 3). In M0 cases, the normal cells showed moderate reactions and the tumor cells showed weak to moderate reactions for IFNAR2.

### IFNAR mRNA expression and survival

**Figure 4:**
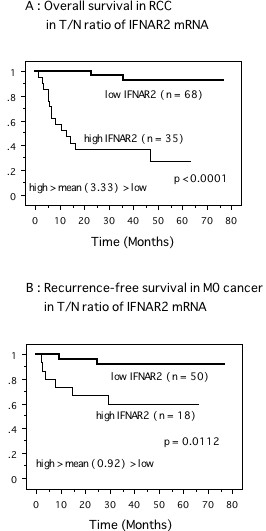
Survival curve in patients with renal cancer based on the mean values of T/N ratio of expression of IFNAR2 mRNAs in kidney tissues, the cases were divided into two groups at this levels – high and low expression. Overall survival curve in total 103 tumors and disease-free survival curve in MO cases of 68 tumors at nephrectomy. *P *value was analyzed by log-rank test.

The mean values for the T/N ratios of IFNAR1 and IFNAR2 mRNAs in the samples were 3.15 (± 1.69) and 3.33 (± 1.15), respectively. As described previously [8,9], the cases were divided into two groups (high and low) based upon whether the expression was above or below this mean. Comparison of the Kaplan-Meier survival rate plots in patients with low vs. high expression of IFNAR2 suggested that a high T/N ratio of IFNAR2 mRNA expression was associated with a shortened overall survival (*P *< 0.0001, Figure 4A). By univariate analysis according to the Cox proportional hazards model, overall survival was influenced significantly by grade, stage, cell type, metastasis, IFNAR1, and IFNAR2 (Table 2). By multivariate analysis, grade, stage, cell type, metastasis, and IFNAR2 were independent prognostic factors (Table 2). Cases of tumors without distant metastasis at nephrectomy (M0; 68 patients) were divided into those with the T/N ratio of IRNAR2 mRNA above or below, a mean of 0.92 (± 0.23). In these localized tumors, Kaplan-Meier plots showed that a high T/N ratio of IFNAR2 mRNA had a significant influence on disease-free survival (*P *= 0.0112, Figure 4B). Although grade, stage, and IFNAR2 predicted disease-free survival in this group in the univariate analyses, stage and IFNAR2 remained significant in the multivariate analysis (Table 2). For IFNAR1, with a mean T/N ratio of 0.85 (± 0.16), there was low expression in 43 patients and high expression in 25 patients, and neither was related to disease-free survival in these patients.

**Table 2:** Cox regression analysis for various potential prognostic factors in survival

		Overall survival (103 patients)					Disease-free survival (68 patients) in M0 at Nephrectomy				
		
Variable	Unfavorable/favorable characteristics	No. of Patients	Analysis	Relative risk	95% confidential interval	P value	No. of Patients	Analysis	Relative risk	95% confidential interval	P value
			Univariate (U)	11.927	4.084 – 34.864	< 0.0001		U	6.357	2.109 – 19.163	0.001
Grade	01/02/2003	32/57/14					17/39/12				
			Multivariate (M)	8.658	1.647 – 45.529	0.0108		M	1.006	0.175 – 5.776	0.9942
			U	8.305	3.430 – 32.714	< 0.0001		U	4.691	2.501 – 13.822	0.002
pT	> 3/2 <	31/72					13/55				
			M	2.171	1.626 – 7.189	0.0225		M	2.447	1.004 – 5.965	0.0489
			U	9.486	3.479 – 25.864	< 0.0001					
Cell type	sarcomatoid/	12/91					0/68				
	acinar		M	2.773	1.302 – 9.587	0.017					
			U	11.798	3.922 – 35.492	< 0.0001					
Metastasis	(+)/(-)	35/68					0/68				
			M	4.362	1.973 – 19.567	0.026					
			U	4.267	1.515 – 12.021	0.006		U	2.028	0.592 – 6.952	0.2605
IFNAR1	high/low	47/56					25/43				
			M	1.958	0.661 – 5.804	0.2252		M	3.244	0.788 – 13.356	0.1031
			U	10.695	4.345 – 26.323	< 0.0001		U	11.03	2.920 – 41.655	0.0004
IFNAR2	high/low	35/68					18/50				
			M	3.813	1.295 – 11.226	0.0151		M	7.604	1.559 – 37.101	0.0121

### IFNAR2 mRNA expression and effect of IFN-α

We also compared the T/N ratio of IFNAR2 mRNA with the effect of IFN-α administration (Figure 1D). Of the M0 cases at nephrectomy (68 patients), the IFNAR2 T/N ratio in the patients with no evidence of disease (NED) in the group receiving IFN-α (M0-IFNa(+)/NED; 0.80 ± 0.24) was higher than those in the untreated group (M0-IFNa(-)/NED; 0.31 ± 0.07, *P *= 0.0267). This result was due to the higher T/N ratio of the M0-IFNa(+)/NED cases with grade 3 and/or pT3/4 cancer. There were no statistical differences in the IFNAR2 T/N ratio between the patients in whom a metastatic lesion appeared (lung, bone, liver, brain, etc.) but was treated with IFN-α (M0-IFNa(+)/Meta; 4.56 ± 1.01) and those left untreated (M0-IFN(-)/Meta; 2.37 ± 0.62). However, the patients with metastatic lesions had a higher T/N ratio of IFNAR2 mRNA in their primary tumors (M0-IFNa(+)/Meta and M0-IFN(-)/Meta) compared with those who had NED after surgery (M0-IFNa(+)/NED and M0-IFNa(-)/NED, *P *< 0.001).

In the M1 cases with only lung metastases at time of nephrectomy (all of conventional acinar type; 23 patients), the IFNAR2 mRNA T/N ratio in the tumors resistant to IFN-α (M1-IFNa(+)/PD; 11.00 ± 2.18) was higher than in those for whom it was effective (M1-IFNa(+)/CR-PR; 1.39 ± 0.22, *P *= 0.0022). Impressively, the M0 patients in whom a metastatic lesion appeared after IFN-α administration (M0-IFNa(+)/Meta) or those in the wait-and-see group and left untreated (M0-IFNa(-)/Meta) had higher IFNAR2 T/N ratios compared to the M1 patients who had a good response to IFN-α (M1-IFNa(+)/CR-PR, *P *= 0.0058, *P *= 0.0233, respectively).

## Discussion

The absolute level of IFNAR mRNAs ranges wildly due to inter-individual variations and is not related to their malignant and metastatic profiles. To take into account the possibility of inter-individual variations in the expression of mRNAs and proteins for IFNAR, we compared the mRNA and protein expressions between paired tumor (T) and non-tumor (N) kidney samples, and the relative yield of the PCR product from the tumor to that from the corresponding non-tumor tissue (T/N) for the expression of IFNAR1 and IFNAR2 mRNAs was calculated as described previously [9,10]. The T/N ratios of IFNAR1 mRNAs were not correlated with any clinicopathologic feature. Impressively, high T/N ratios of IFNAR2 mRNA expression in tumors were associated with poor differentiation, local invasion, metastasis, shortened survival, and a poorer response to IFN-α treatment. The protein levels of IFNAR2c in the primary tumors with synchronous metastases were higher than in those without metastases. To our knowledge, this is the first report analyzing the relationship between IFNAR and renal cancer.

A previous report found no relationship between type 1 IFNAR expression levels in clear cell RCC tumors and stage, grade, gender, age or survival [15]. However, our observation that the relative value of the T/N ratio but not the absolute level of IFNAR2 mRNA in tumor tissues correlated with the malignant and metastatic profiles of RCCs is puzzling even if there is inter-individual variations and should be investigated in future studies. The IFNAR2 mRNA T/N ratio did not correlate with gender or age.

The prognosis of RCCs with a sarcomatoid component is poorer than those without such changes (conventional acinar type), and sarcomatoid RCC is highly resistant to immunotherapy [1]. In this study, 12 patients with a sarcomatoid component presented with metastases at diagnosis, and their prognosis was very unfavorable in comparison to those with a conventional acinar type of RCC. Of the M1 cases, the IFNAR2 mRNA T/N ratio in the sarcomatoid component (13.22 ± 4.89) was higher than that in the conventional acinar type (23 patients; 7.22 ± 1.67, P < 0.01, data not shown). Within the non-sarcomatoid conventional acinar types, the IFNAR2 mRNA T/N ratio in the M1 cases (7.22 ± 1.67) was higher than that in the M0 cases (0.92 ± 0.23, P < 0.0001, data not shown). Moreover, the IFNAR2 mRNA T/N ratio was associated with a poorer prognosis not only in overall survival in all cases but also in disease-free survival in the M0 cases. Therefore, it appears that IFNAR2 is associated with an aggressive and high metastatic potential of the cancer cells. On the other hand, although there was no statistical difference in the IFNAR1 mRNA T/N ratio between the sarcomatoid and conventional acinar types of RCC, a higher IFNAR1 T/N ratio was associated with a poorer overall survival by univariate analysis. This phenomenon may be influenced by the poorer survival of sarcomatoid types. In fact, the IFNAR1 mRNA T/N ratio had no effect on disease-free survival in the M0 cases of non-sarcomatoidal conventional acinar RCC; however, the possible role of IFNAR1 in a larger number of sarcomatoid RCC cases should be examined.

In this study, a higher IFNAR2 mRNA T/N ratio was associated with certain metastatic profiles in RCCs. It is important to try to distinguish between metachronous bilateral RCC from a metastasis in the contralateral kidney. A patient underwent a radical left nephrectomy for a 6 cm, grade 2 RCC at another hospital in 1995. In 2000 at age 78 years, the patient also underwent a partial right nephrectomy with a 1 cm surgical margin for another 1.5 cm, grade 1 RCC tumor, and the IFNAR2 mRNA T/N ratio in the resected tissue was 13.3. The patient was followed closely without receiving any adjuvant immunotherapy. In 2002, a metastatic tumor was found that involved the thyroid gland, and we determined that it also had a high level of IFNAR2 mRNA expression (1.13 fold more than the second primary tumor). Since our data indicate that the IFNAR2 mRNA T/N ratio in grade 1 and pT1a non-sarcomatoid RCCs is very low, while the T/N ratio in this patient's second renal tumor was very high, we believe that the second renal tumor might have been a metastasis from the left kidney to the right. Accordingly, our data suggest that even if conventional pathologic studies indicates a low grade and stage RCC, cases with a high IFNAR2 mRNA T/N ratio need to be followed closely and that the T/N value of IFNAR2 mRNA might be a prognostic factor.

The most commonly used therapy for patients with advanced RCC has been combined immunotherapy employing interleukin (IL)-2 and IFN-α in the USA, while IFN-α is the main agent used in Japan. The question of who obtains a benefit from immunotherapy with IFN-α is an important and unanswered question. Although prognostic factors have recently been recognized to aid in this decision [16], reliable data on immunotherapy using IFN-α in patients with a poor prognosis, in particular for the prediction of recurrence in localized RCCs, are lacking. Recently, Motzer et al. [17] identified five prognostic factors (Karnofsky performance status, time from diagnosis of RCC to treatment with IFN-α, serum lactate dehydrogenese, corrected serum calcium, and hemoglobin) that correlated with overall survival in patients with metastatic RCC treated with IFN-α as their initial systemic therapy. Thus the prognosis of our patients are consistent with Motzer's model. Furthermore, there is evidence that adjuvant IFN-α for localized disease is not recommended unless in specific studies [18]. However, some patients with grade 3/pT2 M1 tumor had a good response to IFN-α (M1-IFNa(+)/CR-PR), while an other patient with a grade 1/pT1 M1 tumor presented with lung metastases and had resistant disease (M1-IFNa(+)/PD) (Table 1). In our analysis regarding the relationship between the IFNAR2 mRNAs T/N ratio and the efficacy of IFN-α immunotherapy, higher T/N ratios correlated with not only the likelihood of metastasis and unfavorable prognosis but also a poorer response to IFN-α even in the non-sarcomatoidal conventional acinar RCCs of lower grade and smaller size (less than 4 cm). These findings might suggest that M0 patients with a higher IFNAR2 mRNA T/N ratio might benefit from being treated with IFN-α to prevent metastasis, and IFN-α would be expected to decrease the tumor burden in M1 patients with a lower T/N ratio. These hypotheses should be studied prospectively in a larger number of patients.

Our data show that there is a upregulation of IFNAR2 mRNAs and IFNAR2c proteins in tumor cells in M1 cases relative to M0 cases. Upregulation of IFNAR2 in tumor cells compared to normal cells in the involved kidney might be advantageous for the anti-cancer effect of IFN-α; however, there have been no published reports of a direct relationship between IFNAR2 expression levels and the effect of externally administered IFN-α. The present study did not make clear whether upregulation was the cause or the result of metastasis. We hope our forthcoming study will clarify this issue.

IFN-α transmits its signals through its homologous receptor complex IFNAR, which is composed of at least two subunits, IFNAR1 and IFNAR2 [5]. IFNAR2 has been reported to be the main ligand-binding subunit [5]. In this study, the IFNAR2 mRNA T/N ratio correlated with the metastatic profiles in RCC. Because of alternative splice variants of IFNAR2 mRNA, we confirmed the actual expression pattern of IFNAR2 by Western blotting analysis. Western blotting analysis showed that the expression level of IFNAR2c protein was higher in the primary tumors in comparison with the non-tumor tissues in the M1 cases but not in M0 cases, indicating that the main component of IFNAR2 expression was the long form of IFNAR2c and that IFNAR2c is the important functional protein associated with the progression of renal cancer. Recent studies have revealed that the activation of STAT3 and AKT (protein kinase B) are associated with progression and poor survival in RCCs [19,20]. It has also been reported that IFNAR2c is responsible for STAT activation [21,22]. Furthermore, these molecules may play key roles in cell survival and proliferation in a large number of human cancers [23]; thus, this pathway may be a molecular target for cancer therapy. We should elucidate the role of this pathway in RCCs in a future study.

Our study results are not sufficient for evaluating the utility of IFNAR2 mRNA T/N ratios as a predictable index for the effect of IFN-α immunotherapy. However, our findings that higher T/N ratios were associated with early recurrence in organ confined tumors and a poorer response to IFN-α administration suggest that analyzing the IFNAR2 mRNA T/N ratios might be useful for predicting prognosis. Although our follow-up period is too short to draw definitive conclusions and setting a cut-off value for the IFNAR2 mRNA ratio in order to select patients for IFN-α immunotherapy may be controversial, we are going to start a prospective study to assess the utility of IFNAR2 mRNA T/N ratios as a predictor of response to IFN-α immunotherapy in patients with RCC. A positive result would substantiate our observations and strengthen their clinical value.

## Conclusion

In this study, the relative value of T/N but not the absolute level of IFNAR2 mRNA, and IFNAR2c protein in tumor tissues correlated with the malignant and metastatic profiles of RCC.

## Competing interests

The author(s) declare that they have no competing interests.

## Authors' contributions

TK and YY initiated the study, participated in its design and coordination, carried out the study, performed the statistical analysis, and drafted the manuscript. KA, HA, and TY carried out the study. MK and KY participated in the design of the study and helped to draft the manuscript. All authors read and approved the final manuscript.

## Pre-publication history

The pre-publication history for this paper can be accessed here:


